# Assessing Matched Normal and Tumor Pairs in Next-Generation Sequencing Studies

**DOI:** 10.1371/journal.pone.0017810

**Published:** 2011-03-18

**Authors:** Liang Goh, Geng Bo Chen, Ioana Cutcutache, Benjamin Low, Bin Tean Teh, Steve Rozen, Patrick Tan

**Affiliations:** 1 Cancer and Stem Cell Biology Program, Duke-National University of Singapore Graduate Medical School, Singapore, Singapore; 2 Department of Medical Oncology, National Cancer Centre Singapore, Singapore, Singapore; 3 Department of Epidemiology and Public Health, National University of Singapore, Singapore, Singapore; 4 Neuroscience and Behavioral Disorders Program, Duke-National University of Singapore Graduate Medical School, Singapore, Singapore; 5 Cellular and Molecular Research/National Cancer Centre Singapore-Van Andel Research Institute Translational Cancer Research Laboratory, National Cancer Centre, Singapore, Singapore; 6 Laboratory of Cancer Genetics, Van Andel Research Institute, Grand Rapids, Michigan, United States of America; 7 Cancer Science Institute of Singapore, Singapore, Singapore; 8 Genome Institute of Singapore, Singapore, Singapore; Memorial Sloan Kettering Cancer Center, United States of America

## Abstract

Next generation sequencing technology has revolutionized the study of cancers. Through matched normal-tumor pairs, it is now possible to identify genome-wide germline and somatic mutations. The generation and analysis of the data requires rigorous quality checks and filtering, and the current analytical pipeline is constantly undergoing improvements. We noted however that in analyzing matched pairs, there is an implicit assumption that the sequenced data are matched, without any quality check such as those implemented in association studies. There are serious implications in this assumption as identification of germline and rare somatic variants depend on the normal sample being the matched pair. Using a genetics concept on measuring relatedness between individuals, we demonstrate that the matchedness of tumor pairs can be quantified and should be included as part of a quality protocol in analysis of sequenced data. Despite the mutation changes in cancer samples, matched tumor-normal pairs are still relatively similar in sequence compared to non-matched pairs. We demonstrate that the approach can be used to assess the mutation landscape between individuals.

## Introduction

Mutations are hallmark of cancers and identification of the mutations is imperative in our understanding of the disease. The advance in next generation sequencing (NGS) has transformed the way to identify mutations, just like microarray did a decade ago. Currently, the technology offers a powerful and yet relatively cost effective approach to characterize genome-wide mutations that occur in diseases. It enables identification of somatic mutations, including base substitutions, indels, chromosomal rearrangements, copy number alterations, and transcriptional aberrations. The rapid increase in NGS publications recently illustrated the potential of the technology, reporting rare mutations in various cancers, many previously undetected [1], [2], [3], [4], [5], [6], [7]. In some of the studies, matched normal-tumor pairs were utilized to identify germline and rare somatic mutations [4], [6], [7], while in other studies, matched tumor-tumor (e.g. primary versus metastatic) were used [1]. While the approach in NGS has been rigorous in the generation of data as well as analysis, we note that there is a lack of quality check to assess if the matched normal-tumor pair was indeed matched. Since identification of germline and de novo rare somatic mutations relies on the matched normal sample, there is necessity to perform quality check on the sequenced data, to ensure the basis of the assumption is upheld. Relatedness check is an essential procedure in linkage and association studies to prevent sample mislabeling or misspecification of relationships between DNA samples which can lead to spurious or biased results [8], [9]. As most of the NGS studies so far involve only one matched pair [3], [6], [7], it would thus seem trivial to check for relatedness. There is however a growing trend towards sequencing large number of samples [4], and given the anticipation that the cost for NGS will drop further, it will soon be possible to do so. It is thus timely to consider relatedness checks and incorporate it as a quality control protocol for NGS analysis.

We propose here, a simple method using the concept of identity-by-state (IBS), an allele sharing approach used in genetics study to verify matched pairs. IBS is often used in single nucleotide polymorphism (SNP) data to ascertain relationship between individuals in the absence of pedigree structure [10]. It measures the degree to which related individuals share alleles and is often used to map complex traits in human relative pairs. Besides relatedness, IBS can also identify meiotic crossovers, and other broad range of chromosomal anomalies such as hemizygous deletions, and uniparental disomy, as well as population structure in families [11]. As IBS has been traditionally used in genetics studies to identify variants in populations, it is thus novel to apply the idea in cancer samples, especially in this particular context of assessing matched normal-tumor pairs. Using IBS on two datasets of matching gastric cancer samples (SNP6 and NGS), we show that clustering of matched normal-tumor samples can be used to assess the ‘matchedness’ of pairs. We also show how IBS can be used to reveal the diversity and occurrence of mutation across the samples.

## Results and Discussion

Two datasets were used in the study; 82 matched pairs on SNP6 and 7 pairs sequenced using NGS (see Methods). Five matched pairs were common in SNP6 and NGS. As IBS is traditionally used in SNP data, we included SNP6 as a validation for the approach.

### Matched and unmatched pairs are clustered differentially

**Figure 1:**
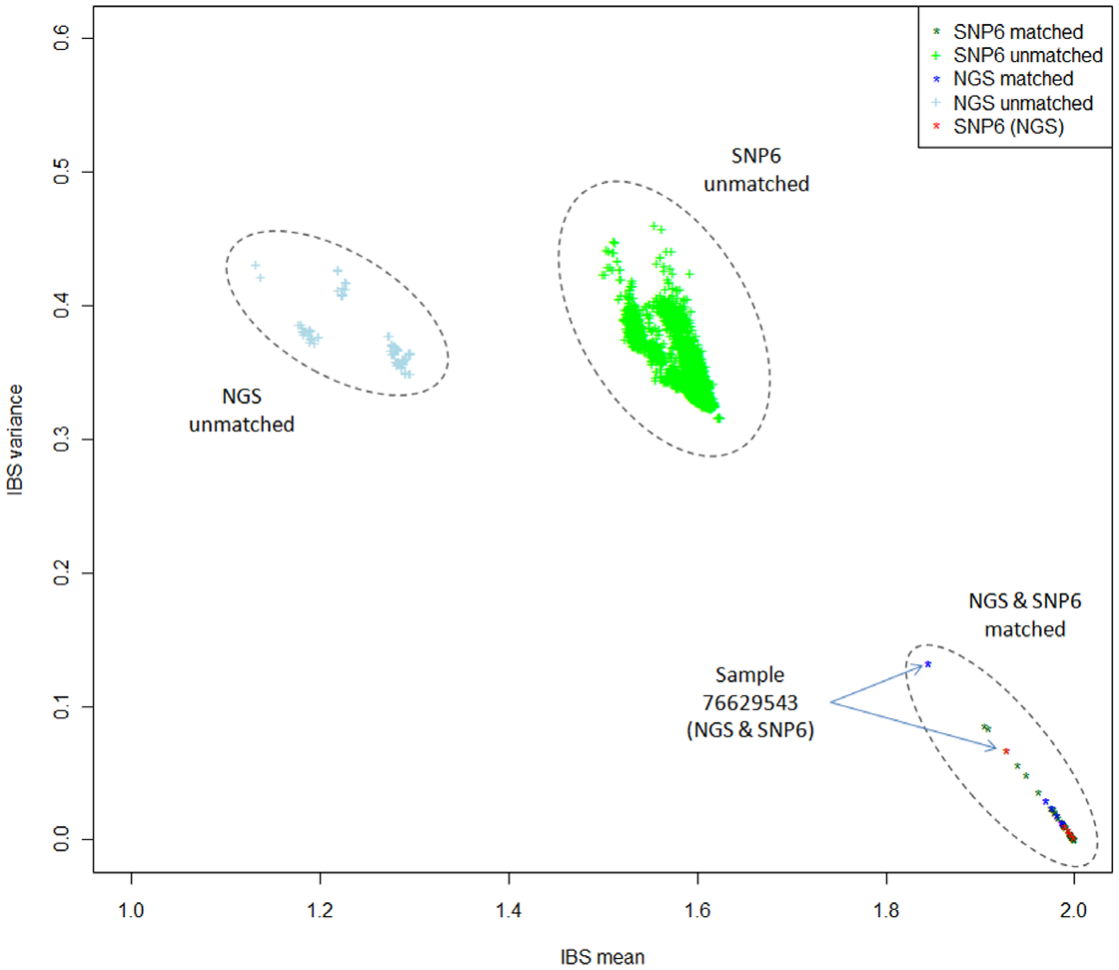
IBS clustering of SNP6 (green) and NGS (blue) samples. IBS for each pair of samples is computed and the mean (x-axis) and variance (y-axis) plotted in the IBS-space. Matched (denoted as *) and unmatched pairs (denoted as +) are clustered differentially where the matched pairs are positioned towards the bottom-right corner indicating more relatedness between samples. 5 SNP6 samples (red) were also sequenced in NGS. One of the samples, 76629543, is clustered further away from the bottom right in both datasets, indicating its higher level of mutations.

Figure 1 showed the clustering of SNP6 and NGS data in the IBS-space which is based on the mean and variance of IBS between two samples. For each dataset, the matched pairs were differentially clustered from the unmatched pairs, and the matched pairs tend to be clustered in the bottom-right corner. This is expected since the relationship of matched samples can be likened to replicates or monozygotic twins, where both alleles are similar. In this scenario, IBS distribution should exhibit a mean near to 2 and with small variance. The basis of this assumption is supported by several studies indicating somatic mutation rate in cancers varies from 1.8 to 3.9 per Mb [2], [12], suggesting that matched pairs will exhibit sufficient ‘replicate-like’ characteristics for differential clustering in IBS. As evident in the clustering of Figure 1, matched pairs were clustered together and further from the unmatched pairs, indicating the differences in the relatedness between the samples. The differential clustering in IBS-space can thus be used to infer matchedness of samples. In this case, it showed that all the samples in both datasets were matched.

From both the SNP6 and NGS data, we observed that although there is differential clustering between the matched and unmatched pairs, the relative position in both data differed slightly. In the ‘NGS & SNP6 matched’ cluster (Figure 1), SNP6 data showed higher IBS mean and lower IBS variance, indicating that mutations in common variants was relatively less compared to NGS, an observation in line with the understanding that SNPs in the Affymetrix SNP 6.0 chip were supposedly well validated common polymorphisms. It is of note that NGS nucleotides were reference variants including dbSNP and novel single nucleotide variants (SNV) targeted mostly at exons (see Methods). As there is uneven distribution of mutation across the genome, with lower prevalence observed in gene regions [7], the level of similarity observed in NGS may be an under estimation of mutations in the samples. Nevertheless, even with the limited exome sequence of NGS or common variants in SNP6, matched tumor-normal pairs can be differentiated using IBS.

Another interesting aspect of the IBS clustering is the range in which matched pairs were spread across a spectrum. For example, sample 76629543 was clustered further away from the other matched samples and bottom right corner. The spread of data points within each cluster indicated the degree of relatedness which may be useful in assessing tumor content. Intuitively, matched samples that are further away from bottom-right would suggest more mutations in the tumor samples. This is also dependent on the quality of the normal samples. We devised a simple IBS_mv_ score (IBS_mean_/IBS_var_) and ranked it against tumor content assessed by pathologist (see Table S1). Using an arbitrary IBS_mv_ of 430 as a cut-off on the SNP6 data, the Wilcoxon test on the distribution of tumor content between the low and high IBS_mv_ groups was 0.0081, indicating that low IBS_mv_ was associated with higher tumor content. The result is indicative but needs to be further validated. Along the same line of thought, IBS can be a means to select samples for sequencing; such as choosing those that are likely to exhibit more mutations, i.e. samples with lower IBS_mv_. This is useful for samples with available SNP data, where IBS can provide a quick analysis to assess the level of mutation.

### Distribution of IBS across genome for matched pairs reveals the mutation landscape

**Figure 2:**
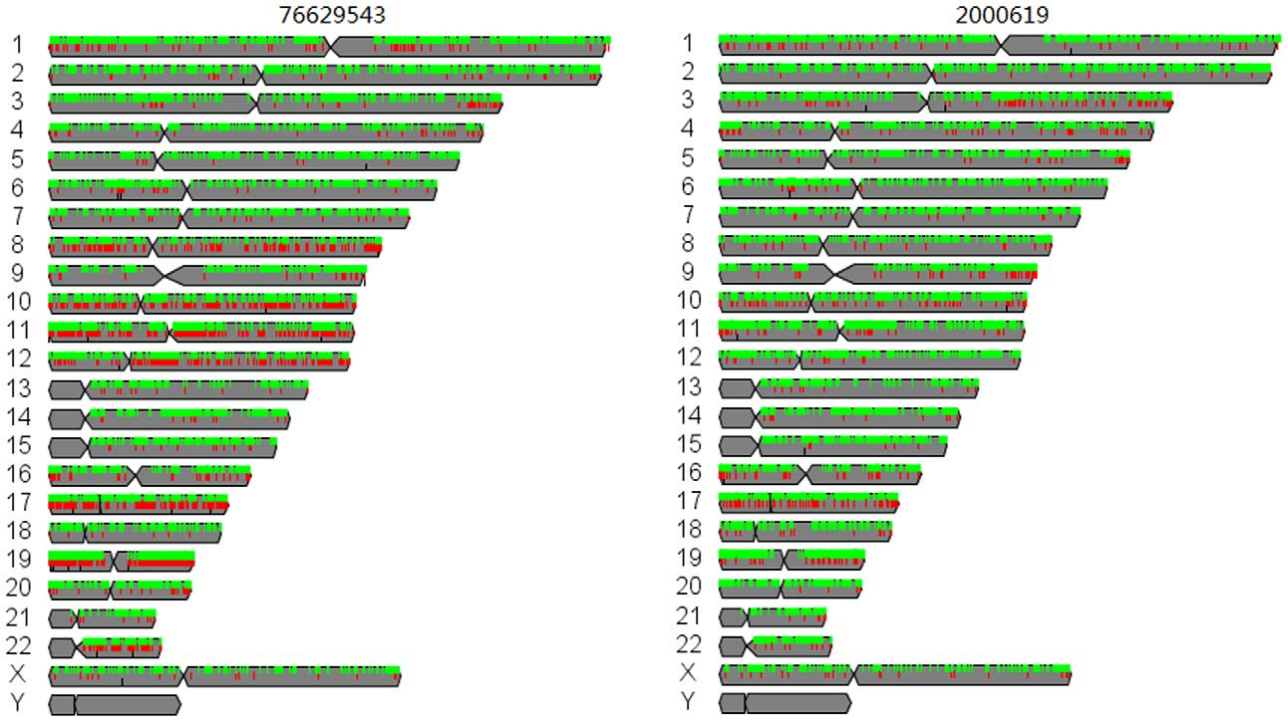
IBS landscape of samples 76629543 and 2000619 in NGS. For each chromosome, the different states of IBS is shown (green: IBS-2, red: IBS-1, black: IBS-0). Sample ID is indicated at the top of each genome plot. Most of the alleles do not change state between matched pairs, i.e. IBS-2 (green). The most frequent allele change is IBS-1 or heterozygous variant (red). IBS-0 or homozygous variant (black) is not common, occurring less than 2%. Sample 76629543 shows the most varied IBS in chr8, 10, 11, 12, 17, 19, and 22q.

Figure 2 showed the IBS of 2 matched pairs (samples 76629543 and 2000619) in NGS across the genome. (For IBS landscape of all 7 matched pairs, see Figure S1.) It revealed the distribution of IBS across the samples, where IBS of 0, 1, and 2 were denoted as IBS-0, IBS-1, and IBS-2 (see Methods). Most of the IBS were IBS-2 as shown by the green ticks in the figure, indicating that both alleles between matched normal and tumor were similar. The frequency of IBS-1 and IBS-0 varied amongst the samples, ranging from 1.2 to 15% (out of all SNVs for each matched tumor-normal pair). Sample 76629543 had the most allele changes in chr8, 10, 11, 12, 17, 19, and 22q, and was the sample clustered furthest away from the matched pairs cluster (Figure 1). The diversity in its IBS landscape was in concordance with its low IBS_mv_. Table 1 summarized the frequency of IBS in the 7 NGS samples. Most of the changes were IBS-1 which were heterozygous variants; i.e. AA/BB->AB (somatic) or AB->AA/BB (LOH). Frequency of LOH varied from 37.4% to 90.6%. Homozygous variant (i.e. IBS-0, AA->BB) on the other hand was not common, occurring less than 2% of all IBS-1 and IBS-0, mostly in dbSNP. Of the 7 samples, more than 3 samples had IBS-0 or IBS-1 in chr6 (HLA-A, HLA-B, HLA-C), chr10 (FANK1, TUBGCP2), chr17 (CDC27), and chr22 (CYP2D6). There were 53 IBS-0 of which 2 samples has IBS-0 in ADAMTS9. The most IBS-1 for LOH was found in KIR3DP1 and RYR1 (2 samples with 15 LOH each). Detailed analysis of the variants is still in progress and will be reported elsewhere.

**Table 1:** IBS-0 and IBS-1 (somatic and LOH) frequency summary of the NGS samples.

Sample	AA/BB->AB (Somatic, IBS-1)	AB->AA/BB (LOH, IBS-1)	AA->BB (IBS-0)	Total
990172	151 (50.84)	143 (48.15)	3 (1.01)	297
990300	311 (57.06)	224 (41.1)	10 (1.83)	545
990355	203 (61.7)	123 (37.39)	3 (0.91)	329
990475	170 (50.6)	163 (48.51)	3 (0.89)	336
2000619	220 (29.69)	515 (69.5)	6 (0.81)	741
2000778	260 (56.03)	201 (43.32)	3 (0.65)	464
76629543	318 (8.76)	3287 (90.55)	25 (0.69)	3630

Percentages are indicated in parenthesis.

### Conclusion

One of the underlying assumptions in this approach is the relatively low mutation rate in cancer. This was shown by our own NGS study on gastric cancer kinome [13] as well as others who looked at a broad range of tumors. Although it is known implicitly that mutations are a hallmark of cancers, we have not yet quantified the similarity or dissimilarity between a cancer genome and the matched normal. Surprisingly, the difference is relatively small, such that non-matched samples, whether between normal or tumors, are still considerably more different than matched normal-tumor. In this paper, we demonstrated that clustering in IBS-space provides a robust quantitative approach to assess matchedness of paired samples. A novel metric IBS_mv_ may be used to assess tumor content as well as selection of samples for sequencing using SNP data. In addition, the IBS landscape offers a genomic view of the mutation across the samples. As shown, the mutations were mostly heterozygous somatic or LOH. Homozygous variants were less common and most found in this study were in dbSNP.

## Methods

Primary and matched gastric tissues were obtained from the Singhealth Tissue Repository, after approvals from Institutional Research Ethics Review Committees, and with signed patient informed consent. Samples were isolated from patients and characterized by pathology. Tumor content information was available for 22/82 samples in SNP6 and 1/7 in NGS (see Table S1).

### SNP6

Genomic DNA was hybridized to the Affymetrix SNP 6.0 chip, following the manufacturer's instructions. The raw data was normalized with normal as controls using Affymetrix Genotyping Console. 82 matched samples were available for this study. To maintain compatibility with NGS dataset, 30K SNPs were selected randomly across the 22 autosomes to generate the SNP6 dataset. To check for bias in the selection of SNPs, several SNP data consisting of 20K and 25K randomly selected SNPs were generated and the IBS computed using our algorithm and the visual tool graphical relationship representation (GRR) [10] (see Figure S2). GRR is a visual tool using IBS to assess relatedness in pedigree. IBS was also computed for the entire SNP dataset (see Figure S3). The plots showed similar clustering regardless of number of SNPs. The genetic tool Plink [14] was used to generate the various SNP datasets in this study.

### NGS

Seven matched pairs (5 from the 82 samples in SNP6) were sequenced using array-based sequence capture (Agilent SureSelect) and Illumina GAIIx sequencer, targeting mostly the exons. The data was put through our sequencing pipeline consisting of base calling using the Illumina Pipeline, mapping and alignment with BWA [15], PCR duplication removal with SAMTools [16], and GATK [17] for variants calling. To incorporate the matchedness check as a quality control, we analyzed all the variants from reference sequence (dbSNP and novel) output from GATK with consensus quality ≥30, read depth ≥5, and variant depth ≥2. On average, there are 24857 SNVs per sample, 24427 SNVs between matched pairs and 32286 SNVs between unmatched pairs. For comparison, GRR was also used to assess the IBS clustering (see Figure S4).

### IBS computation

Any two individuals (or samples), whether related or not, can share 0, 1, or 2 alleles, denoted as IBS-0 (both alleles are different), IBS-1 (one of the allele is different), and IBS-2 (both alleles are the same) respectively. Alleles in the dataset were coded using A and B and pairwise IBS was computed between all samples. See Table 2 for the possible scenarios between samples' alleles and IBS score. Display of the IBS landscape was done using GenomeRelator (http://www.chromosomechronicles.com/2009/10/22/identity-by-state-snp-analysis-find-relatives-test-paternity-and-determine-allele-sharing/). The R code for IBS computation is available at http://research.duke-nus.edu.sg/papers/IBS.zip.

**Table 2:** IBS scores between samples.

Sample 1	Sample 2	IBS
AA	AA	2
AA	AB	1
AA	BB	0
AB	AB	2
AB	BB	1
BB	BB	2

## Supporting Information

Figure S1 **IBS landscape of 7 matched pairs in NGS.**
(TIF)

Figure S2 **Comparison of IBS for 20K, 25K, and 30K SNPs using our algorithm (left) and GRR (right).** Note that GRR shows standard deviation (y-axis) instead of variance. Clustering in both plots is similar regardless of the number of SNPs indicating that there is no bias and a smaller set of SNPs would suffice for assessing matched tumor-normal pairs.
(TIF)

Figure S3 **IBS plot of all 868155 SNPs.** The clustering is similar to the datasets of 20K, 25K, and 30K SNPs indicating that the clustering is not bias by number of SNPs. Note that this computation was not possible with GRR and IBS computation for entire SNP dataset can be intensive even with our algorithm.
(TIF)

Figure S4 **IBS plot using GRR showed similar clustering. Note that y-axis is standard deviation instead of variance in the manuscript.**
(TIF)

Table S1 IBS scores and tumor content for the 22 samples in SNP6 data. ^*^Sample 76629543 was sequenced (NGS) and profiled for SNP6.
(XLSX)
