# Supplementary figures and images for: Assessing Matched Normal and Tumor Pairs in Next-Generation Sequencing Studies
Source: PLoS One. 2011 Mar 18;6(3):e17810. doi: 10.1371/journal.pone.0017810 (PMC3060821; doi:10.1371/journal.pone.0017810)

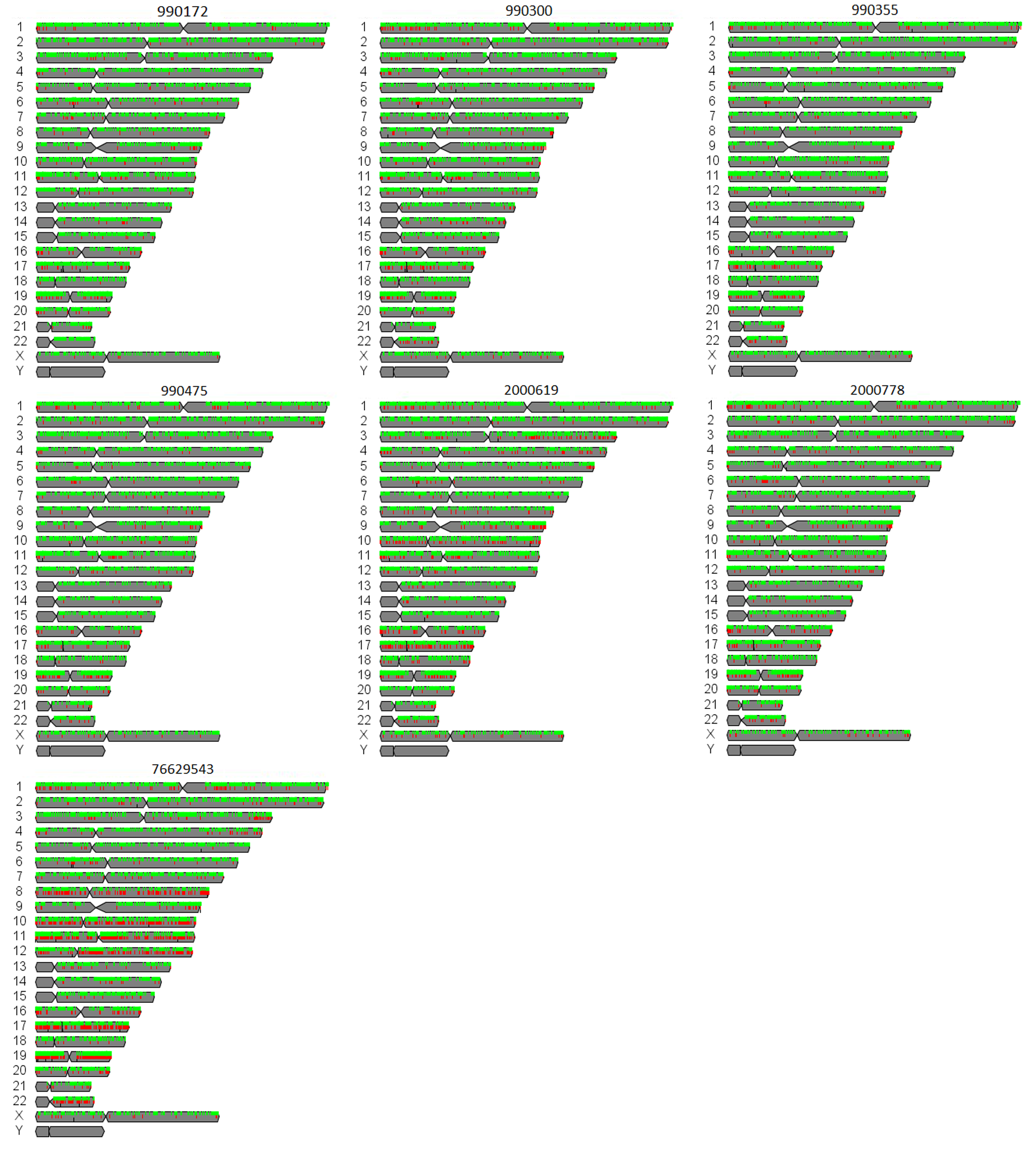

Supplement: Figure S1 — IBS landscape of 7 matched pairs in NGS. (TIF) [file pone.0017810.s001.tif]

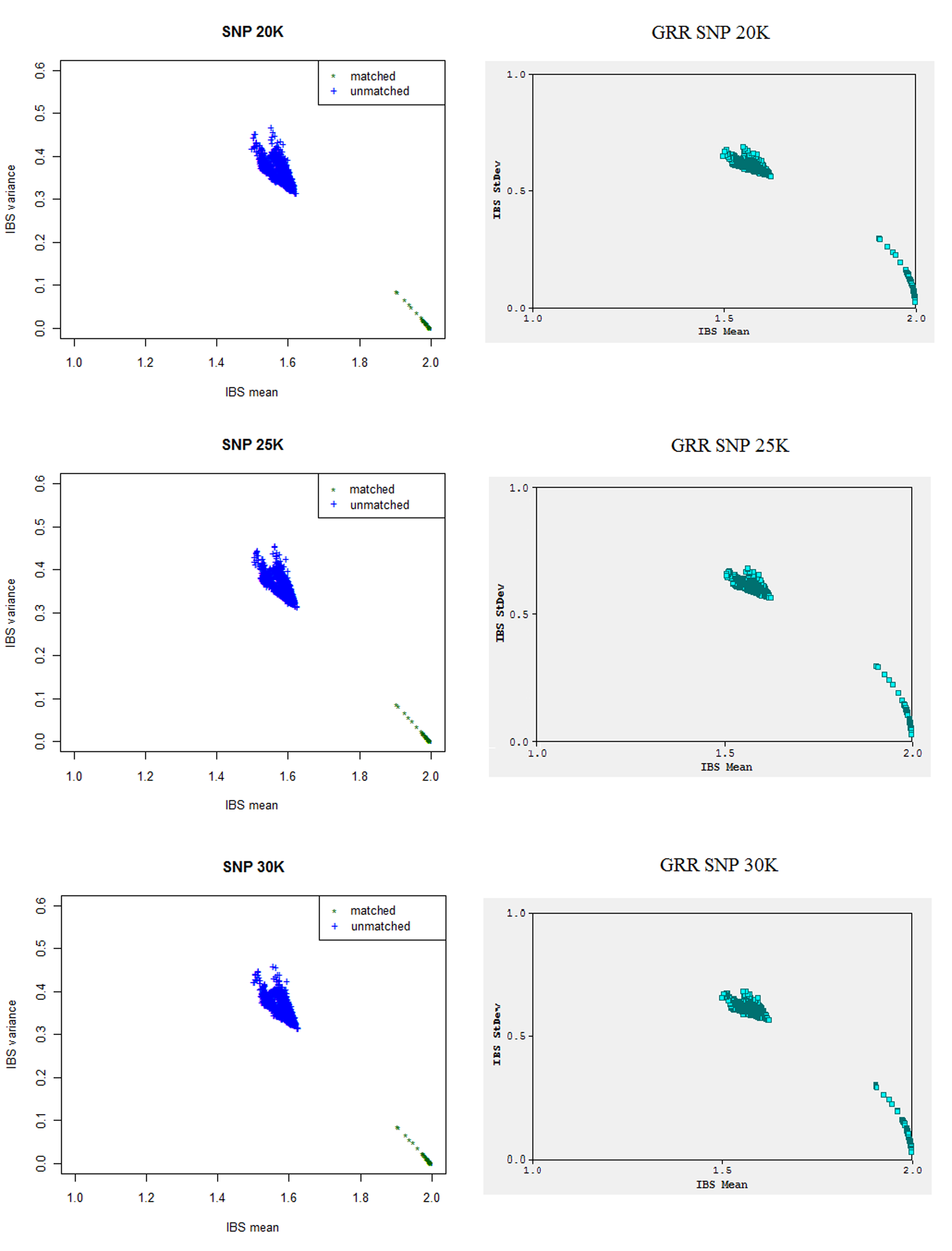

Supplement: Figure S2 — Comparison of IBS for 20K, 25K, and 30K SNPs using our algorithm (left) and GRR (right). Note that GRR shows standard deviation (y-axis) instead of variance. Clustering in both plots is similar regardless of the number of SNPs indicating that there is no bias and a smaller set of SNPs would suffice for assessing matched tumor-normal pairs. (TIF) [file pone.0017810.s002.tif]

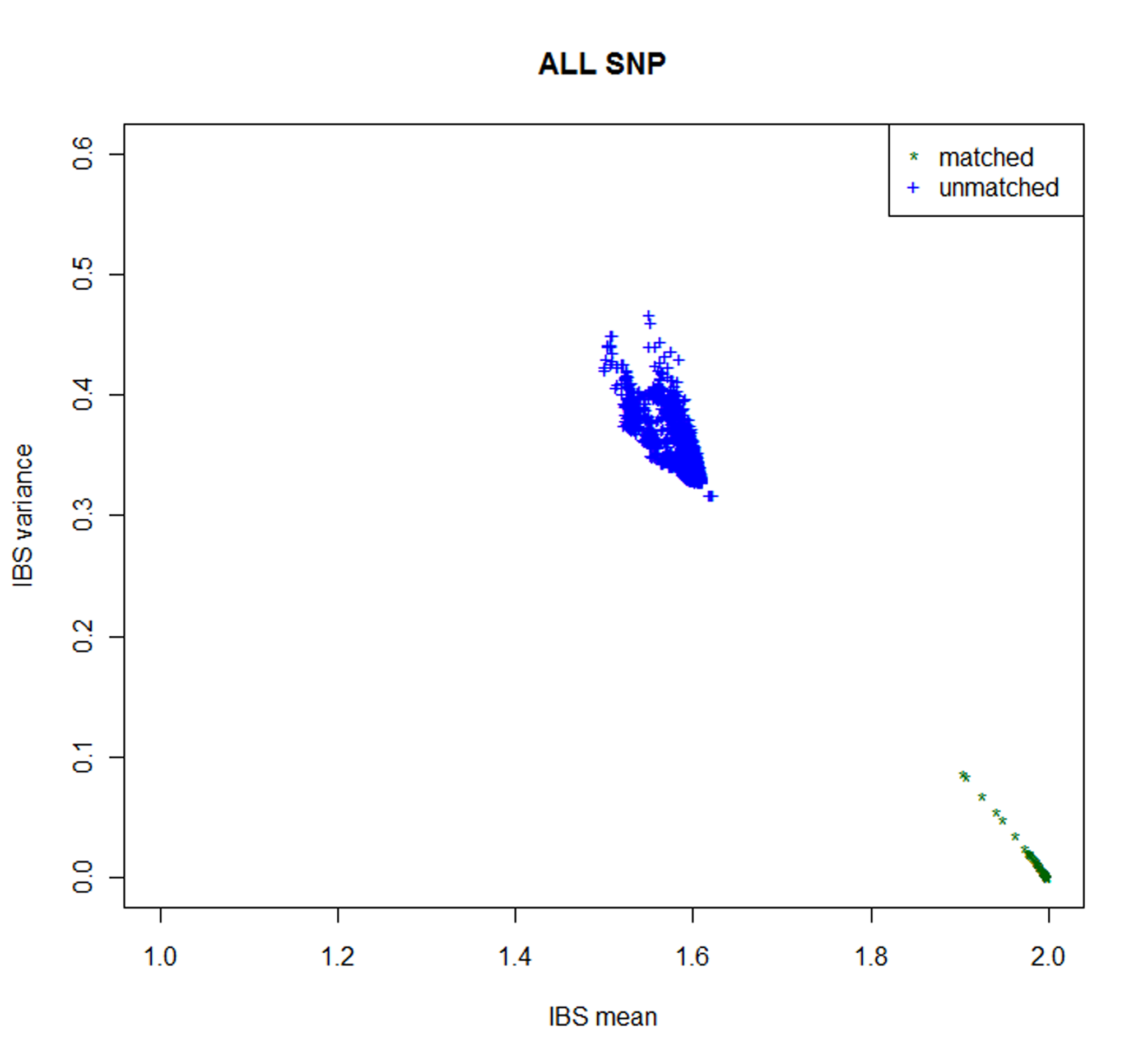

Supplement: Figure S3 — IBS plot of all 868155 SNPs. The clustering is similar to the datasets of 20K, 25K, and 30K SNPs indicating that the clustering is not bias by number of SNPs. Note that this computation was not possible with GRR and IBS computation for entire SNP dataset can be intensive even with our algorithm. (TIF) [file pone.0017810.s003.tif]

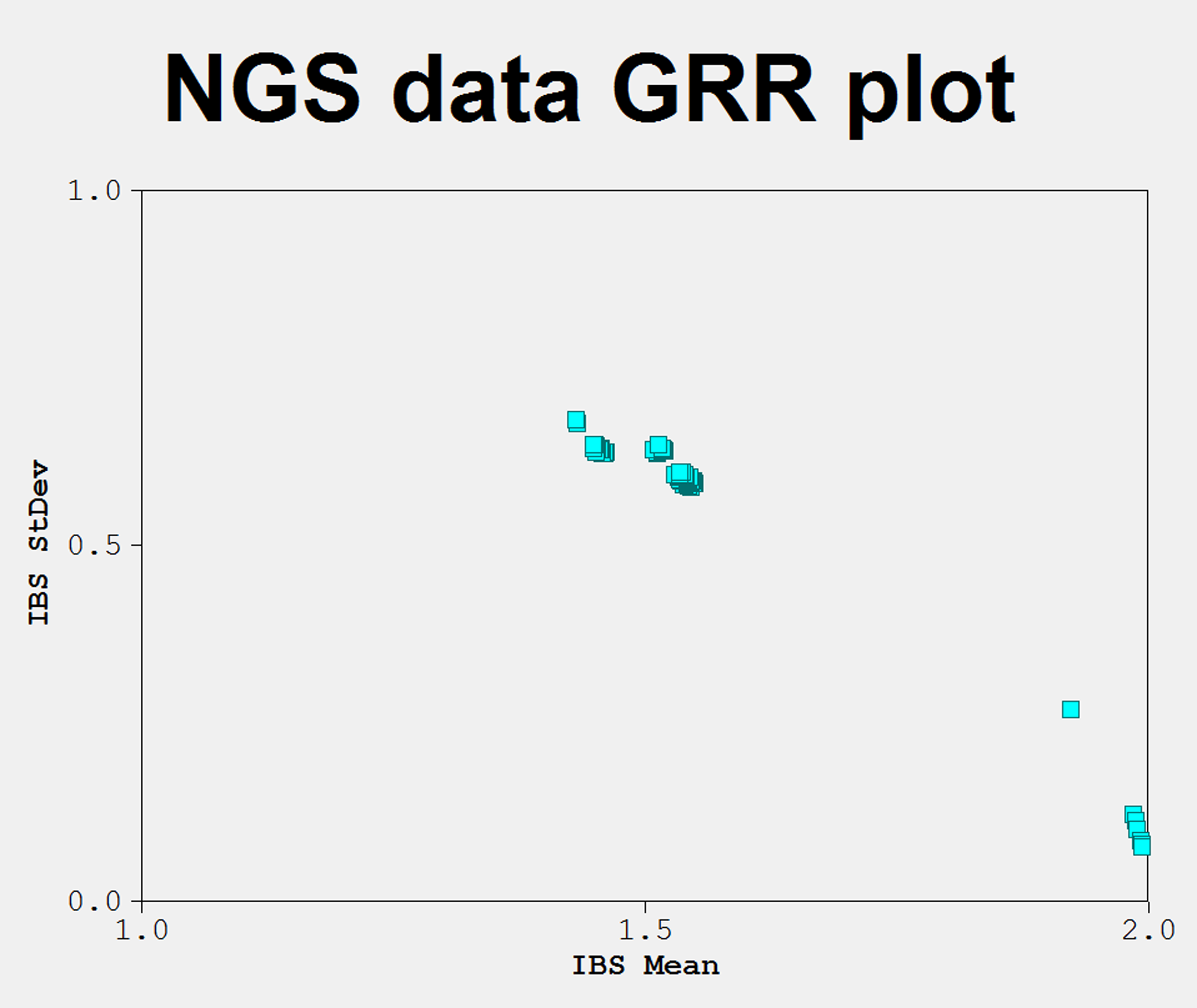

Supplement: Figure S4 — IBS plot using GRR showed similar clustering. Note that y-axis is standard deviation instead of variance in the manuscript. (TIF) [file pone.0017810.s004.tif]
